# Complexity analysis of fNIRS signals in ADHD children during working memory task

**DOI:** 10.1038/s41598-017-00965-4

**Published:** 2017-04-11

**Authors:** Yue Gu, Shuo Miao, Junxia Han, Ke Zeng, Gaoxiang Ouyang, Jian Yang, Xiaoli Li

**Affiliations:** 1 Institute of Electrical Engineering, Yanshan University, No. 438, Hebei Street, HaiGang District, Qinhuangdao 066004 China; 2 Children’s Hospital Attached to The Capital Institute of Paediatrics, No. 2, Yabao Street, ChaoYang District, Beijing 100020 China; 3 State Key Laboratory of Cognitive Neuroscience and Learning & IDG/McGovern Institute for Brain Research, Beijing Normal University, No. 19, Xinjiekou Wai Street, Haidian District, Beijing 100875 China

## Abstract

Attention-deficit/hyperactivity disorder (ADHD) is a prevalent neurodevelopmental disorder in children. Neuroimaging studies have revealed abnormalities of neural activities in some brain regions, including the frontal cortex, striatum, cerebellum, and occipital cortex. Recently, some investigators have demonstrated that nonlinear complexity analysis of neural activity may provide a new index to indicate ADHD. In the present study, we used the permutation entropy (PE) to measure the complexity of functional near-infrared spectroscopy (fNIRS) signals in children with and without ADHD during a working memory task, it was aimed to investigate the relationship between the PE values and the cortical activations, and the different PE values between the children with and without ADHD. We found that PE values exhibited significantly negative correlation with the cortical activations (*r* = −0.515, *p* = 0.003), and the PE values of right dorsolateral prefrontal cortex in ADHD children were significantly larger than those in normal controls (*p* = 0.027). In addition, the PE values of right dorsolateral prefrontal cortex were positively correlated to the ADHD index (*r* = 0.448, *p* = 0.012). These results suggest that complexity analysis of fNIRS signals could be a promising tool in diagnosing children with ADHD.

## Introduction

Attention-deficit/hyperactivity disorder (ADHD) is one of the most prevalent neurodevelopmental disorders, which is characterized by difficulty paying attention, excessive activity, and impulsivity. Generally, ADHD arises in childhood and frequently persists into adolescence and adulthood^1–3^. It is estimated that about 5–8% of school-aged children are affected by ADHD^4^. Consequently, children with ADHD have difficulties controlling their behaviors or paying their attentions, which result in an adverse effect on academic performance and social function^1^. Furthermore, this disorder may increase a risk of other disorders which are not directly related to ADHD, such as antisocial disorders^5, 6^. Therefore, it is important to explore valid biomarkers for earlier diagnosis and intervention.

Working memory is defined as the temporary storage and manipulation of information^7, 8^, which has been considered as a core deficit in ADHD^9^. Some subsequent neuropsychological measures have proposed to detect working memory deficits in youth and adults with ADHD^10, 11^. These impairments in ADHD include the performance on digit span, consonant trigrams and mental arithmetic^12^. N-back task is a common working memory paradigm, which has been used in many functional neuroimaging researches on ADHD. Functional magnetic resonance imaging (fMRI) data from ADHD adults showed significantly decreased activity in cerebellar and occipital regions and a trend toward decreased activation in prefrontal cortex compared to control subjects during a 2-back task^12^. Besides, the activation of parietal cortex was also decreased in ADHD patients during a verbal n-back task^13^. The study of electrophysiological activation during the visual n-back found that frontal theta event-related synchronization was significantly reduced in ADHD compared to control subjects, meanwhile, ADHD patients showed lower alpha event-related desynchronization and higher subsequent alpha event-related synchronization^14^. These studies indicate that n-back working memory tasks are associated with prefrontal function^12^.

Recently functional near-infrared spectroscopy (fNIRS) has been applied in the neuroscience, which is a non-invasive optical imaging technique that can measure the concentration changes in oxygenated hemoglobin ([HbO]) and deoxygenated hemoglobin ([Hb]) associated with functional brain activity^15^. The subjects need to wear a cap embedded with detector-emitter pairs of near-infrared light, meanwhile they are without the constraints of confined space. One detector-emitter pair generates one channel. This form makes fNIRS better to localize the neural signals than EEG. In addition, fNIRS is more low-cost and more robust to movement than fMRI. With its relatively low cost, portability, and high ecological validity, fNIRS is particularly suitable for investigating the brain function of children and patients with ADHD^16, 17^.

Many previous studies of fNIRS about ADHD mainly focus on the abnormalities of brain activation during different experimental paradigms. In the studies of subjects performing a Stroop task, which requires the inhibition of competing response, controls had a significant increase in [HbO] over right dorsolateral prefrontal cortex, whereas the ADHD children did not^18, 19^. The Go-NoGo task requires individuals to inhibit prepotent response. During the NoGo block, ADHD children had a weaker increase in [HbO] over the prefrontal cortex compared to controls, which indicated that ADHD children were not activating the prefrontal cortex^20^. In working memory processes, adult patients with ADHD had a reduced lateral prefrontal activation during n-back task^21^. These studies consistently indicated hypoactivity in the prefrontal cortex in ADHD patients.

It is well known that the human brain is a complex nonlinear system^22^. Output neurophysiologic signals exhibit complex fluctuations both in spatial and temporal^23^, which reflect nonlinear dynamical processes^24^. Consequently, nonlinear analysis was applied to neurophysiologic signals for providing a novel understanding of the complexity of human brain activity and the physiological processes in either healthy or pathological populations^25, 26^. Several complexity analysis methods were reviewed in Takahashi’s paper^24^. In the past few decades, novel nonlinear approaches based on entropy have been widely used to measure the complexity of physiological signals. In general, entropy methods describe the degree of underlying randomness of a random signal^27^. Random signal with large entropy has a high level of randomness, in contrast, random signal with small entropy corresponds a low level of randomness^27, 28^. Entropy methods have been successfully applied in many areas, such as depth of anesthesia monitoring^29, 30^, seizure prediction^31^, and mental disorders^24^. For example, in studies about ADHD, the sample entropy of EEG signals in the alpha frequency band decreased in ADHD children during a multi-source interference task^22^, and the adult patients with ADHD showed reduced sample entropy of fMRI signals in frontal and occipital regions during resting state^32^. These results were consistent with the hypothesis that there is a loss of complexity in the dynamics of many integrated physiological processes with disease^33, 34^. However, sample entropy is heavily dependent on the data length and may result bias for short data sets^35^. Moreover, sample entropy is sensitive to parameter choices, especially the selection of similarity criterion^36^. Permutation entropy (PE) as an appropriate complexity measure for time series can overcome these issues^37^. PE statistically measures the different orders of neighboring relative values in the time series, rather than estimating the similarity like sample entropy. Therefore, it does not need to set the similarity criterion. According to the report of Bandt and Pompe, PE is robust to the choice of data length (128 samples are sufficient)^37^. Moreover, PE is a normalized value, which makes the comparison between different results more easily. Therefore, PE may provide a novel tool to investigate neural signals with ADHD.

In this study, our aim was to investigate the relationship between the complexity of fNIRS signals and the cortical activation, and the change of brain complexity in ADHD children and controls during an n-back task. To address these issues, we measured the fNIRS signals of both ADHD children and normal controls during a digital n-back task. We used the general linear model (GLM) to detect the activation areas in the both groups during the task. PE was applied to measure the complexity of fNIRS signals. Then, the differences between the PE values of the two groups were further statistically evaluated. Finally, Pearson’s correlation coefficient was used to test the relationship between the PE values and the cortical activations.

## Results

### Behavioral performance

The data of reaction time (RT) and accuracy (ACC) were listed in the Table 1. We used two-sample t-test to compare the performance data between the two groups in each condition. There were no significant differences between the two groups on any of the task performance data. However, there was a trend for the controls to perform faster and more accurately than the ADHD children. Although the trend of the results was consistent with the previous studies^12, 38, 39^, the control and ADHD groups in this study showed poorer performances on the n-back task than those in previous studies. We think the difference of age may be the main reason why the subjects showed poorer performances in this study. The subjects in this study are younger than those in previous studies.

**Table 1:** Behavioral performances.

	Controls Mean (SD)	ADHD Mean (SD)	*p* value
1-back RT (ms)	908 (195)	988 (215)	*p* = 0.29
1-back ACC (%)	70.7 (16.1)	60.1 (20.7)	*p* = 0.12
0-back RT (ms)	732 (147)	833 (214)	*p* = 0.13
0-back ACC (%)	89.9 (10.7)	81.2 (16.2)	*p* = 0.09

### Activation analysis

**Figure 1:**
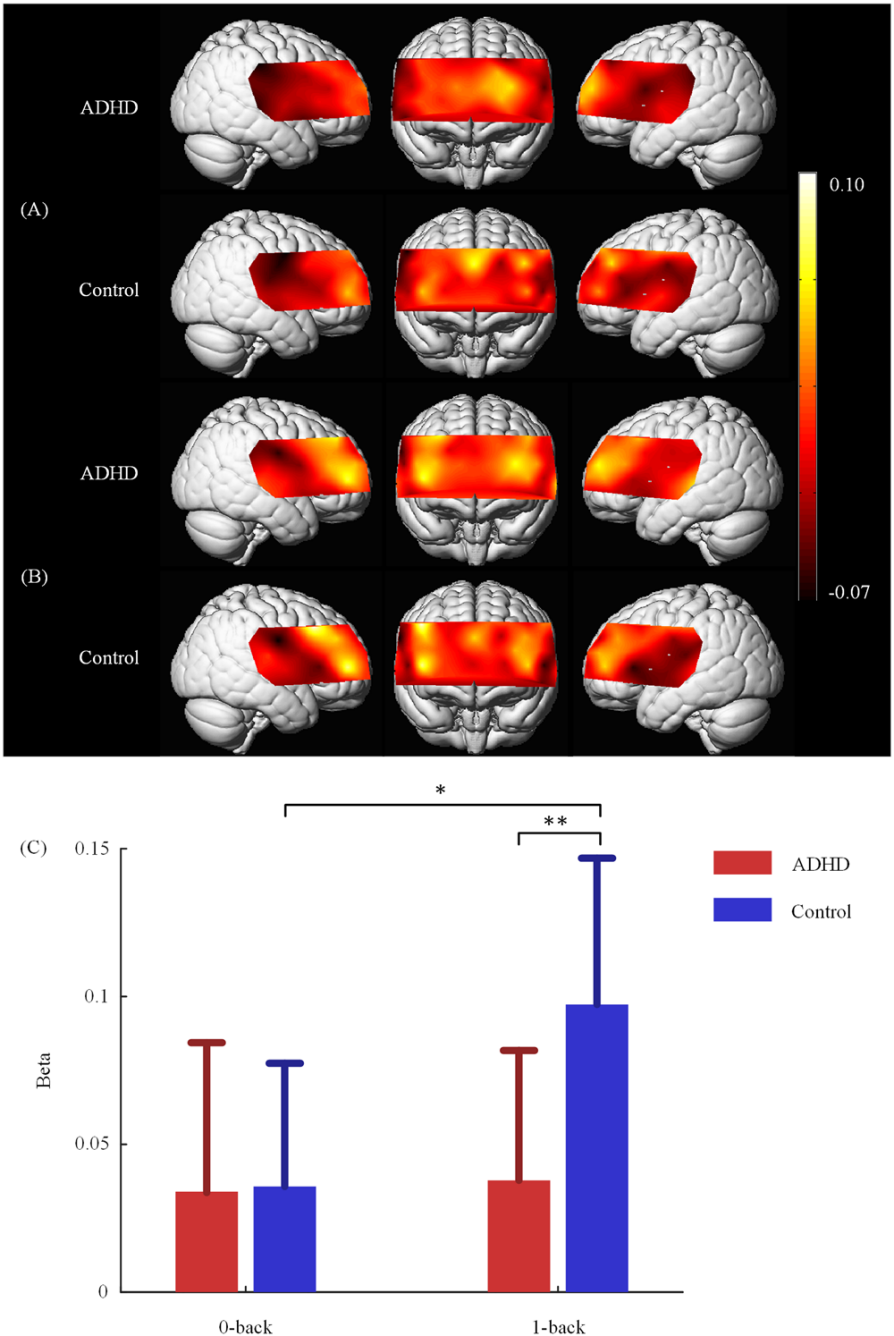
fNIRS activation maps for 0-back condition (**A**) and 1-back condition (**B**). The beta values are indicated by color. The light colors represent strong cortical activations, and vice versa. Bar plots of beta values showing the beta values of channel 3 in each conditions for the ADHD and control group (**C**). Error bars represent the standard error of the mean. *p < 0.05; **p < 0.01.

We analyzed the activation levels in each condition within each group. The distributions of activation levels in each condition within each group were shown in Fig. 1(A,B). There were no regions in which the ADHD group activated significantly in any condition. There were also no significant differences between the two conditions in ADHD group. As well as the ADHD group, the control group had no significant activations in 0-back condition. However, the control group significantly activated right DLPFC (channel 3) and VLPFC (channel 35) in 1-back condition (*t*_15_ = 5.93, *p* = 0.001; *t*_15_ = 5.17, *p* = 0.006). We then performed a subtractive contrast (1-back minus 0-back) within each group. In the subtractive contrast, the control group had more significant activation in right DLPFC (channel 3) in 1-back condition than in 0-back condition (*t*_15_ = 4.32, *p* = 0.032, see Fig. 1(C)). Differences between the two groups in each condition were also examined. There were no regions in which the control group had greater activation than the ADHD group in 0-back condition. In 1-back condition, the control group more strongly activated right DLPFC (channel 3) than the ADHD group (*t*_29_ = 4.39, *p* = 0.007, see Fig. 1(C)).

### Permutation entropy analysis

**Figure 2:**
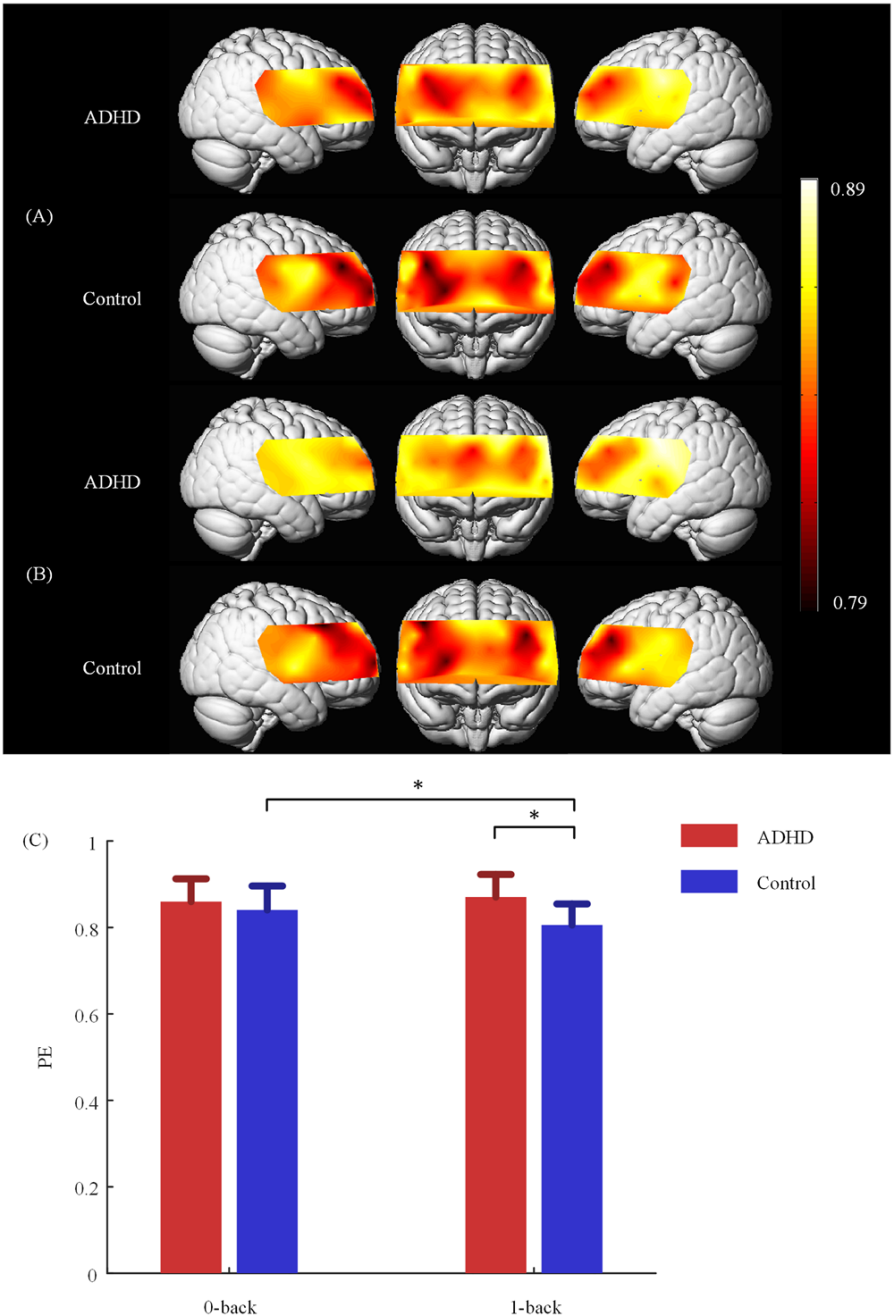
PE maps for the 0-back condition (**A**) and 1-back condition (**B**). The PE values are indicated by color. The light colors represent large PE values (high complexity), and vice versa. Bar plots of PE values showing the PE values of channel 3 in each conditions for the ADHD and control group (**C**). Error bars represent the standard error of the mean. *p < 0.05.

The complexity of fNIRS signals was measured by PE. The larger the value of PE is, the more irregular or complex the time series is, and vice versa. We calculated the PE values in 0-back and 1-back separately. The distributions of PE values in each condition within each group were shown in Fig. 2(A,B). In 0-back condition, the PE values in bilateral DLPFC and right VLPFC were relatively small for the two groups. In 1-back condition, the control group obtained a distribution of PE values similar to they did in 0-back condition. As well as bilateral DLPFC, the PE values in superior MPFC were relatively small for the ADHD group. Then we compared the PE values between the two conditions within each group. There were no significantly different PE values in any region in the ADHD group, although the PE values in right DLPFC, VLPFC and TC were lightly larger, and the PE values in superior MPFC were lightly smaller in 1-back condition than in 0-back condition. In the control group, the PE values of channel 3 in right DLPFC were significantly smaller in 1-back condition than in 0-back condition (*t*_15_ = −4.23, *p* = 0.038, see Fig. 2(C)). We also compared the PE values between the two groups in each condition. In 0-back condition, there were no regions in which the ADHD group obtained significantly different PE values compared to the control group. In 1-back condition, the PE values of channel 3 in right DLPFC were significantly larger in ADHD group (*t*_29_ = 3.91, *p* = 0.027, see Fig. 2(C)).

### Correlation analysis

**Figure 3:**
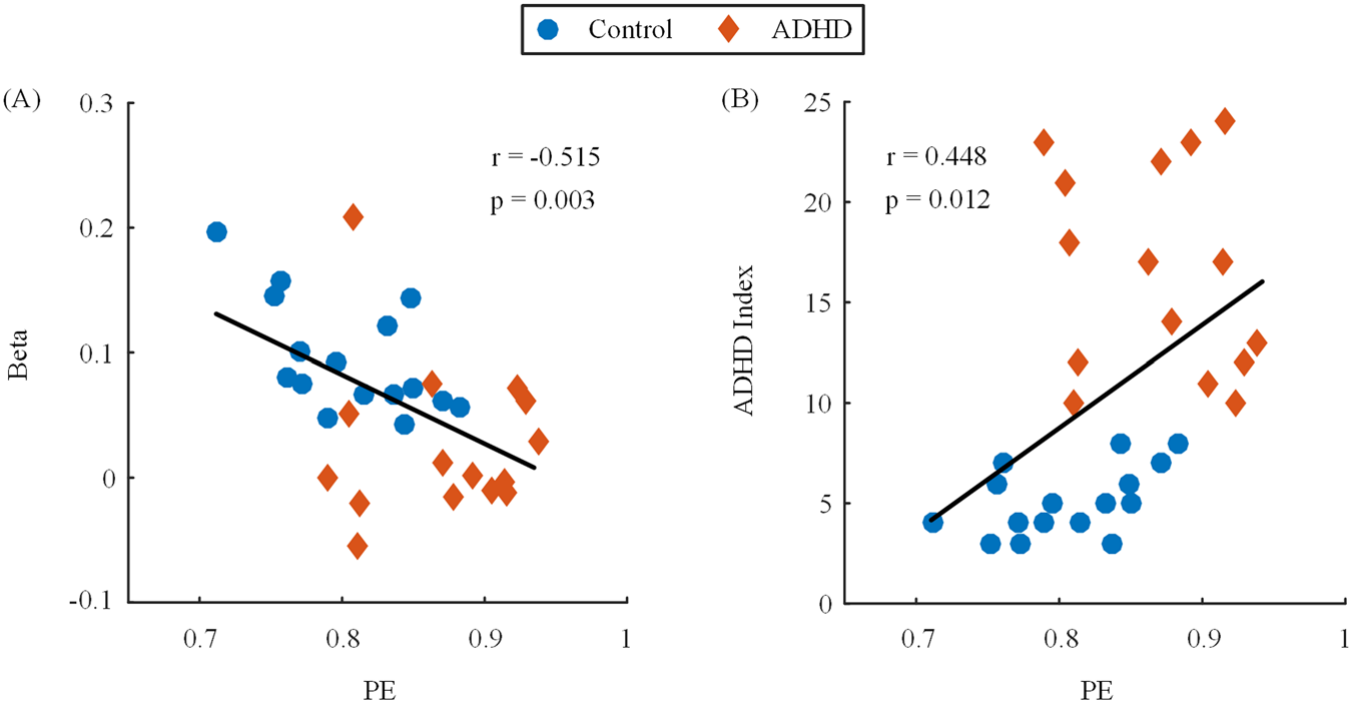
Scatter plots showing the correlation between the PE values and the cortical activations (**A**), and the ADHD index (**B**). The PE values were negatively correlated to the cortical activations ($r=-0.515$, $p=0.003$) and positively correlated to the ADHD index ($r=0.448$, $p=0.012$).

Significant difference in PE values was found in right DLPFC (channel 3). Thus, we used the data of channel 3 in 1-back condition for the correlation analysis. The Pearson’s correlation coefficient was used to test the relationship between the PE values and the cortical activations, and the ADHD index. The results are shown in Fig. 3, in the form of scatter plots. The number of data points in each panel was 31. Each data point in each panel represented one subject. The beta values estimated in GLM were used to represent the activation level. Obviously, the PE values were negatively correlated to the cortical activations (*r* = −0.515, *p* = 0.003), and positively correlated to the ADHD index (*r* = 0.448, *p* = 0.012).

## Discussion

In this study, we used PE to analyze the complexity of fNIRS signals in children with and without ADHD during a working memory task. We investigated the relationship between the complexity (PE values) and the activation patterns, and sought the differences in complexity between fNIRS signals acquired from children with and without ADHD during a working memory task.

The application of complexity analysis to physiological signals has provided important information about cortical abnormalities in neuropsychiatric disorders that may not be apparent by linear analysis. In previous studies, approximate entropy (AE) and sample entropy (SE) have been used for complexity analysis of neural signals in ADHD^32, 40, 41^. However, the disadvantage of AE and SE is that both of them require long, stationary and noiseless data^42^. Fortunately, PE as a new complexity statistical parameter does not have such limitations. This is because PE calculation just considers the order relation between the values of a time series instead of the values themselves. In addition, the value of PE is based on the distribution of ordinal patterns, PE is therefore less sensitive to noise embedded in fNIRS signals. Meanwhile, PE is conceptually simple, computationally efficient and artifact resistant. Therefore, PE is more suitable for application of complexity analysis to fNIRS signals.

In the present study, we found that the control group significantly activated the right DLPFC and VLPFC in 1-back condition than in 0-back condition, and more strongly activated right DLPFC than the ADHD group. This indicates that greater working memory demands evoke greater activations in brain regions associated with working memory, including dorsolateral prefrontal cortex, inferior frontal junction^39^. Additionally, in the ADHD group, there are functional deficits of right DLPFC which is as the main neural correlate of the central executive^43^. The n-back tasks are classical executive function tests in that they require subjects to monitor stimulus and update information in working memory to generate appropriate responses^44^. In a meta-analysis, Smith *et al*. reported that an increasing demand on n-back tasks was associated with increasing DLPFC activity in healthy subjects^45^. As the review of Cubillo *et al*., there are deficits of executive functions in children and adults with ADHD^46^. Whether the structural studies or the functional imaging studies, indicated that the DLPFC which is an important region related to the executive functions differentiate ADHD patients from controls. Shallice *et al*. inferred that the executive function differences between ADHD patients and controls might be associated with a higher-level effort mechanism^47^.

In our complexity analysis we found that the PE values of right DLPFC in ADHD children were significantly larger than those in controls. The global PE values of ADHD children were relatively increased during the working memory task. But they were not significantly different. Generally, if the signal is measured from an inactive region, it should contain more spontaneous brain activity, and thus its PE should reach a relatively high value. If the signal is measured from an active region, it should be more ordered, and thus has a smaller PE^48^. As mentioned above, the ADHD children didn’t activate the right DLPFC due to the functional deficits. This makes the fNIRS signals from the right DLPFC not be modulated by the working memory task in ADHD children. That is to say, the fNIRS signals from the right DLPFC are more random during the working memory task in ADHD children. Therefore, the complexity of corresponding region in ADHD children is higher than that in controls. That is also why the cortical activations and PE values exhibited significantly negative correlation in right DLPFC.

However, our results are different from some previous studies which reported relatively decreased complexity of brain activity in ADHD subjects^32, 49^. We inferred that there may be three main reasons. First, the fNIRS signals we used were recorded from prefrontal cortex and partial temporal cortex, while the signals previous studies used were recorded from the whole brain. According to the hypothesis that there is a loss of complexity in the dynamics of many integrated physiological processes with disease^33, 34^, we think the complexity of other regions we did not measure should be lower in ADHD children compared to controls. Second, we used different neuroimaging modalities during different tasks. We recorded the fNIRS signals during an n-back task, while the previous studies recorded their fMRI or MEG signals at resting state. Third, our subjects are younger. The brain signal complexity of healthy subjects will increase with age^50^, while the brain signal complexity the ADHD patients will not increase due to the neurodevelopmental deficit. These factors combine to cause the results in previous studies different from this study.

We think there are two main advantages of PE analysis over traditional activation analysis. First, the PE analysis has the lower signal-noise-ratio requirements than the traditional activation analysis, because the PE analysis is less affected by the amplitude of the signals and less sensitive to the noise embedded in the signals. Second, PE analysis can be applied to both task state and resting state, while the traditional activation analysis can be just used during a task. In the future, we also hope to investigate the complexity of fNIRS signals during resting state in ADHD children. Note that, the two methods describe different physical phenomena. The PE analysis can not supersede the traditional activation analysis. The PE analysis of fNIRS signals just provides a new research perspective on the ADHD.

Based on this study, we think there are several implications of the PE value. First, the negative correlation between PE value and activation level implies that the PE value may estimate the cortex activation degree during a cognitive task. Second, the difference of the PE values between ADHD group and control group implies that the PE value may be as a parameter of neurofeedback for rehabilitation of ADHD children. Third, the correlation between PE value and ADHD symptom severity implies that the PE value may serve as a potential candidate for neurophysiologic marker of ADHD.

It should be noted that, the results presented in this study might be limited to children samples but not to other age groups. Hence, we should test our results on a larger sample, and consider the effect of age and gender in the further studies. Meanwhile, we just chose the empirical values as the parameters of PE calculation. We should systematically discuss the effect of parameters to PE calculation in the further studies. Furthermore, we also aim to evaluate and compare the complexity of the brain at resting state in the near future studies.

## Conclusion

To our knowledge, this is the first study to investigate the complexity of fNIRS signals in children with and without ADHD during a working memory task. We evaluated the complexity of brain by using the PE metric. It was found that the cortical activations and PE values exhibited significantly negative correlation, and the PE values of right DLPFC in ADHD children were significantly larger than those in controls. The results reveal that the right prefrontal complexity was changed in ADHD, and suggested that ADHD may be related to neural disconnection. Therefore, the complexity analysis based on PE may provide a new view to understand the neural mechanism of ADHD. Although there are some limitations in this study, we still suggest that the complexity analysis based on PE is a promising tool in diagnosing ADHD.

## Materials and Methods

### Participants

Thirty-one children, consisted of fifteen with ADHD (5 females and 10 males, aged 6–9 years, mean ± standard deviation 7.6 ± 1.4) and sixteen normal controls (6 females and 10 males, aged 6–9 years, mean ± standard deviation 7.3 ± 1.3), participated in this study. Children with ADHD were recruited randomly from Capital Institute of Pediatrics’ Children’s Hospital and diagnosed by an experienced child psychiatrist using the diagnostic interview that assesses ADHD according to the DSM-V criteria. The severity of ADHD symptoms was assessed using the ADHD index from the Conners’ rating scales for parents. No ADHD children exhibited any complication such as neurological limitation, epilepsy, mental retardation, or genetic disorder. In addition, all the ADHD children were drug-naive. The controls were recruited from the local community and had no history of any mental or neurological disorders. The intelligence quotients (IQ) of all children were evaluated by means of the Chinese version of the Wechsler Intelligence Scale for Children-Revised (WISC-R). There were no significant differences between the groups in terms of age and IQ. The detailed demographic data of subjects were listed in Table 2. This study was approved by the ethics committee of Children’s Hospital Attached to The Capital Institute of Pediatrics, and all methods were carried out in accordance with the relevant guidelines and regulations. Written informed consent was obtained from the parents of all children.

**Table 2:** Demographic data of subjects.

	Controls Mean (SD)	ADHD Mean (SD)
Number of subjects	16	15
Age	7.3 (1.3)	7.6 (1.4)
IQ	110 (12)	107 (11)
ADHD index	5.1 (1.2)	16.5 (5.1)

#### Experimental protocol

**Figure 4:**
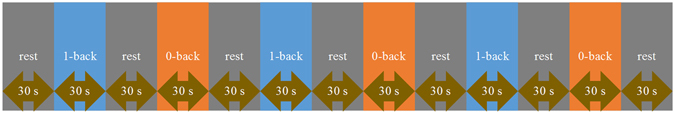
Schematic of the experimental procedure. The order of the task conditions was random across subjects. The adjacent two tasks were separated by 30 s periods of rest. Just one possible order was shown in this figure.

Figure 4 summarizes the experimental procedure. A blocked periodic design that incorporated 0- and 1-back tasks was used in the present study. The order of the task conditions was random across subjects and the participants didn’t know the task order prior to the start of experimentation. The adjacent two tasks were separated by 30 s resting segments during which participants were instructed to sit still and relax. Each of the two conditions was conducted three times. Each block began with 5000 ms presentation of the condition cue (specifying 0- or 1-back). In addition, a 30 s baseline period preceded the first task segment. Within each condition, 10 single digits were pseudo randomly shown to the participants. Each digit was presented for 300 ms, followed by an interstimulus interval of 1700 ms. Every block contained 15 trials. For the 0-back condition, the participants were instructed to press the button under the right index finger whenever a digit that appeared on the computer screen in front of them was identical to the target digit, otherwise, press the button under the right middle finger. For the 1-back condition, the participants had to press the button under the right index finger whenever the presented digit was identical to the preceding one, otherwise, press the button under the right middle finger. For both conditions, a total of 12 target trials appeared. The reaction time (RT) and accuracy (ACC) were recorded for analysis of behavioral data. The n-back task was generated by E-Prime (version 2.0, Psychology Software Tools, Inc., Pittsburgh, PA, USA), and presented in a 17′ computer screen. The distance between the subject’s eyes and the screen was approximately 50 cm. Before the fNIRS measurement, all participants were instructed about the task, and trained to practice some trials of the task. An experimenter observed the course of practice to confirm that the participants understood the paradigm correctly.

### fNIRS measurements and preprocessing

**Figure 5:**
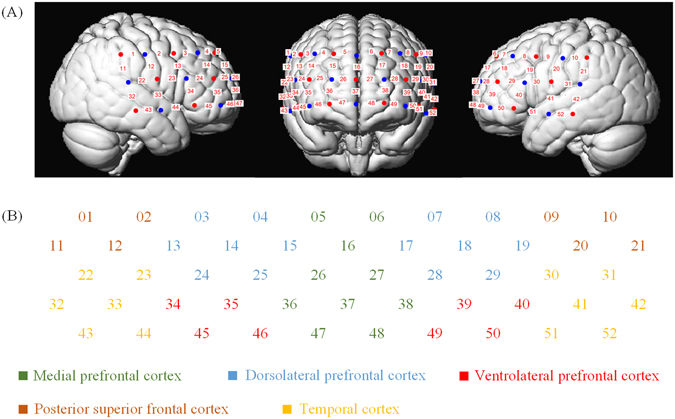
Schematic arrangement of the fNIRS probe array. (**A**) Location of optodes (red = emitters; blue = detectors) and channels (numbers) over a standard brain model. (**B**) Anatomical areas covered by the probe array. Different colors represent different anatomical areas.

In this study, we used the ETG-4000 (Hitachi Medical Company, Japan) NIRS system working with two different wavelengths of near infrared light (695 nm and 830 nm) to measure the [HbO] and [Hb]. The sampling frequency was 10 Hz. We used a “3 × 11” measurement patch, which was placed on the head with regard to the relevant standard positions of international 10–20 system for EEG electrode placement. The middle inferior optode was placed over Fpz and inferior row of optodes was oriented in direction of T3 or T4 respectively (Fig. 5(A)). In this patch, 17 emitters and 16 detectors were positioned in an alternating fashion, forming 52 measurement channels. The emitter-detector distance was 3 cm.

We mapped fNIRS channels to the corresponding areas of a brain model. According to literature^51^, we divided these channels into nine regions as shown in Fig. 5(B) (left VLPFC: 39, 40, 49, 50; right VLPFC: 34, 35, 45, 46; left DLPFC: 7, 8, 17, 18, 19, 28, 29; right DLPFC: 3, 4, 13, 14, 15, 24, 25; left PSFC: 9, 10, 20, 21; right PSFC: 1, 2, 11, 12; left TC: 22, 23, 32, 33, 43, 44; right TC: 30, 31, 41, 42, 51, 52).

The [HbO] and [Hb] were calculated using the modified Beer-Lambert law^52^. Because [HbO] signals can reflect changes in regional cerebral blood oxygenation and provide a better signal to noise ratio^53^, we only used [HbO] data in this study. The motion artifacts in [HbO] data were corrected using the empirical mode decomposition method^54^. To remove the high-frequency physiological noise and low-frequency baseline drift, a band-pass filter (4th order infinite impulse response Butterworth filter) between 0.01 to 0.3 Hz was applied to the [HbO] data. At last, the [HbO] data was down-sampled to 2 Hz.

### General linear model analysis

GLM analysis has been increasingly used to analyze fNIRS data in many studies to identify cortical areas significantly stimulated by given tasks^55^. In this study, we also employed GLM to analyze channel-wise hemodynamic responses in the cortex stimulated by the n-back task. GLM models the measured brain response as a linear combination of predicted responses due to variable stimulations plus an error term, which can be formulated as:

1 $$y={x}_{0}{\beta }_{0}+{x}_{1}{\beta }_{1}+e$$

where *y* is the measured hemodynamic response ([HbO] in this study) at each channel, *x*_0_ and *x*_1_ are the predicted stimulation-evoked responses (0-back and 1-back) which are generated by convolving the task onset with the canonical hemodynamic response function, ${\beta }_{0}$ and ${\beta }_{1}$ are the estimated amplitudes of [HbO], and $e$ is the error term. By using the method of least squares, we would obtain the estimated ${\beta }_{0}$ and ${\beta }_{1}$. Last, cortical activation levels were obtained by statistically analyzing the ${\beta }_{0}$ and ${\beta }_{1}$.

### Permutation entropy

**Figure 6:**
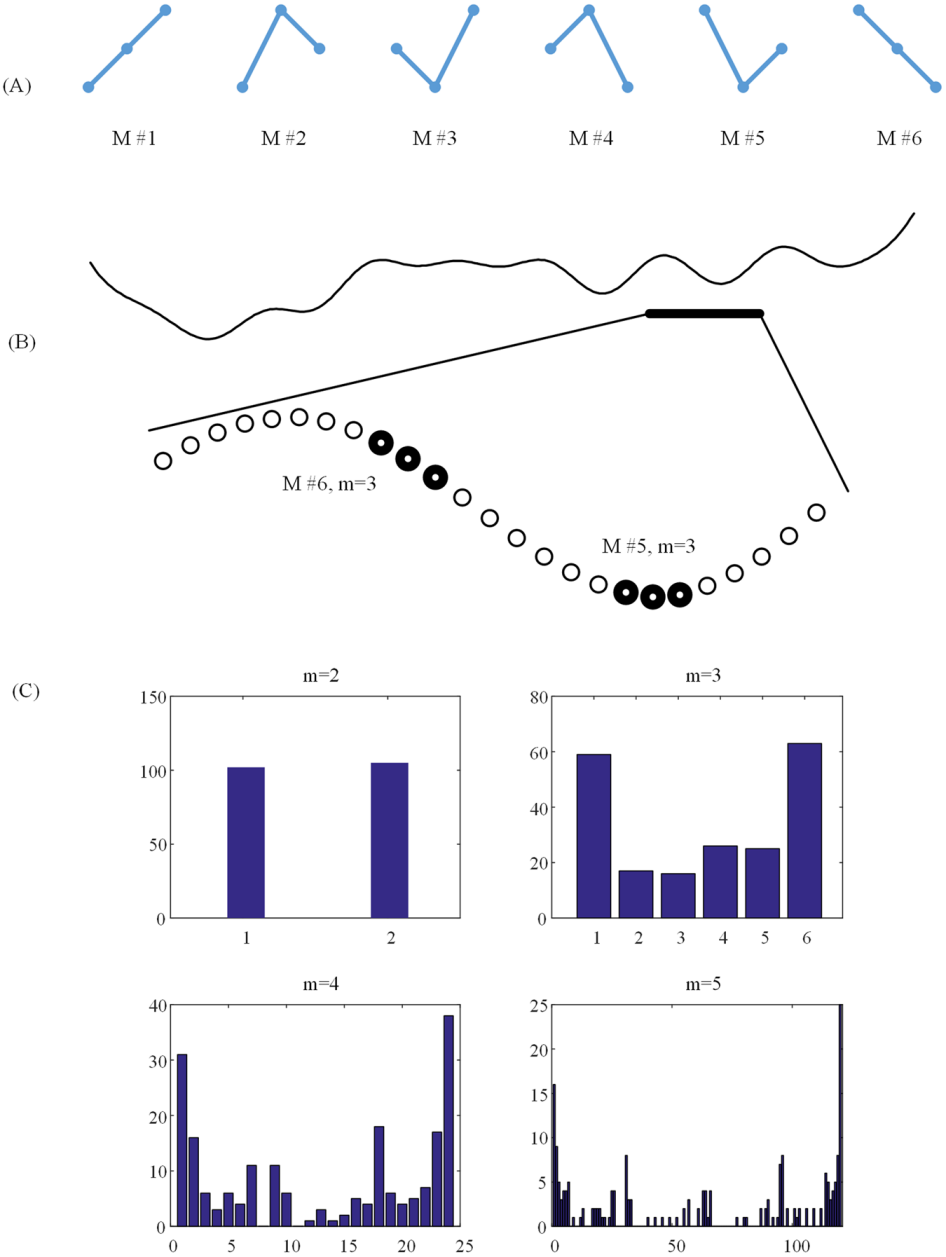
(**A**) Six motifs for the embedding dimension of 3. M represents motif. (**B**) A segment of fNIRS data used in this study. (**C**) The number of every order pattern at $m=2$, 3, 4 and 5. Horizontal ordinate represents the order pattern. Vertical ordinate represents the number of the order pattern.

Permutation entropy was originally proposed by Bandt and Pompe^37^ as a quantitative complexity measure of a dynamical time series. With the PE method, a time series is first mapped to a sequence of order patterns based on comparison of neighboring values, and then PE is the statistical measure of relative frequencies of the different order patterns. Given a time series $\{{x}_{i}:1\le i\le N\}$, vectors ${X}_{i}=\{{x}_{i},{x}_{i+\tau },\ldots ,{x}_{i+(m-1)\tau }\}$, $1\le i\le N-(m-1)$ with the embedding dimension $m$ and time lag $\tau$ are constructed. Then, vector ${X}_{i}$ is arranged in an ascending order: $\{{x}_{i+({j}_{1}-1)\tau }\le {x}_{i+({j}_{2}-1)\tau }\le \ldots \le {x}_{i+({j}_{m}-1)\tau }\}$. For $m$ dimensions, there are $K=m!$ possible order patterns, which are also called motifs. As illustrated in Fig. 6(A), there are six different motifs for $m=3$. Each vector ${X}_{i}$ can be represented by one of the $K$ motifs. The probability of occurrence of the $j$ th motif, ${P}_{j}$, is then calculated. The PE of this time series is defined as:

2 $$PE=-\sum _{j=1}^{K}{P}_{j}\,\mathrm{ln}\,{P}_{j}$$

The corresponding normalized entropy is defined as follows:

3 $$PE=\frac{-{\sum }_{j=1}^{K}{P}_{j}\,\mathrm{ln}\,{P}_{j}}{\mathrm{ln}\,K}$$

The largest value of PE is 1, meaning the time series is random and the smallest value of PE is 0, meaning the time series is absolutely regular. That is, the larger the value of PE is, the more irregular or complex the time series is, and vice versa. The calculation of PE is dependent on the selection of embedding dimension $m$. If $m$ is too small ($m=1$, or 2), only very few distinct order patterns are included in the time series, so this can’t work. If $m$ is too large ($m > 10$), it is not sure that every possible motif occurs in the time series. As shown in Fig. 6(C), when $m=2$, the number of the two order patterns were almost equal, however, when $m=4$ or $m=5$, some order patterns didn’t occur in our data. Therefore, according to the characteristics of fNIRS data, we think $m=3$ is appropriate.

### Statistical analysis

For the performance data, two-sample t-test was used for the between-group comparisons. In activation analysis, within-group analysis was performed using one-sample t-test for 0-back contrast, 1-back contrast and subtractive (1-back minus 0-back) contrast; between-group comparisons were completed using two-sample t-test. In PE analysis, the PE values in each condition (0-back and 1-back) were calculated channel by channel for each subject, and the two-sample t-test was performed to identify the significantly different regions between the two groups. Since there are 52 channels, we can calculate 52 values (beta or PE). The t-tests were performed repeatedly across 52 channels. The Bonferroni correction was then applied to correct for multiple comparisons. The p-value × 52 < 0.05 is considered as significant result. The p-value × 52 was reported in this study. The Beta-maps and the PE-maps for both groups were generated by a custom software based on NIRS-SPM^55^ and SPM-fNIRS (https://www.nitrc.org/projects/spm_fnirs/). Last, we used the Pearson’s correlation coefficient to test the relationship between the PE values and the cortical activations, and the ADHD index.

## References

[1] Wang L (2009). Altered small-world brain functional networks in children with attention-deficit/hyperactivity disorder. Hum Brain Mapp.

[2] Monden Y (2015). Individual classification of ADHD children by right prefrontal hemodynamic responses during a go/no-go task as assessed by fNIRS. Neuroimage Clin.

[3] Rosenberg MD (2016). A neuromarker of sustained attention from whole-brain functional connectivity. Nat Neurosci.

[4] Bush G (2010). Attention-deficit/hyperactivity disorder and attention networks. Neuropsychopharmacology.

[5] Monden Y (2012). Clinically-oriented monitoring of acute effects of methylphenidate on cerebral hemodynamics in ADHD children using fNIRS. Clin Neurophysiol.

[6] Mannuzza S, Klein RG, Bessler A, Malloy P, Hynes ME (1997). Educational and occupational outcome of hyperactive boys grown up. J Am Acad Child Adolesc Psychiatry.

[7] Baddeley A (1996). The fractionation of working memory. Proc Natl Acad Sci USA.

[8] Cubillo A (2014). Drug-specific laterality effects on frontal lobe activation of atomoxetine and methylphenidate in attention deficit hyperactivity disorder boys during working memory. Psychol Med.

[9] Barkley RA (1997). Behavioral inhibition, sustained attention, and executive functions: constructing a unifying theory of ADHD. Psychol Bull.

[10] Kovner R (1998). Neuropsychological testing in adult attention deficit hyperactivity disorder: a pilot study. Int J Neurosci.

[11] Castellanos FX, Tannock R (2002). Neuroscience of attention-deficit/hyperactivity disorder: the search for endophenotypes. Nat Rev Neurosci.

[12] Valera EM, Faraone SV, Biederman J, Poldrack RA, Seidman LJ (2005). Functional neuroanatomy of working memory in adults with attention-deficit/hyperactivity disorder. Biol Psychiatry.

[13] Kobel M (2009). Effects of methylphenidate on working memory functioning in children with attention deficit/hyperactivity disorder. Eur J Paediatr Neurol.

[14] Missonnier P (2013). EEG anomalies in adult ADHD subjects performing a working memory task. Neuroscience.

[15] Jobsis FF (1977). Noninvasive, infrared monitoring of cerebral and myocardial oxygen sufficiency and circulatory parameters. Science.

[16] Ishii-Takahashi A (2014). Prefrontal activation during inhibitory control measured by near-infrared spectroscopy for differentiating between autism spectrum disorders and attention deficit hyperactivity disorder in adults. Neuroimage Clin.

[17] Nagashima M (2014). Acute neuropharmacological effects of atomoxetine on inhibitory control in ADHD children: a fNIRS study. Neuroimage Clin.

[18] Jourdan Moser S, Cutini S, Weber P, Schroeter ML (2009). Right prefrontal brain activation due to Stroop interference is altered in attention-deficit hyperactivity disorder - A functional near-infrared spectroscopy study. Psychiatry Res.

[19] Negoro H (2010). Prefrontal dysfunction in attention-deficit/hyperactivity disorder as measured by near-infrared spectroscopy. Child Psychiatry Hum Dev.

[20] Inoue Y (2012). Reduced prefrontal hemodynamic response in children with ADHD during the Go/NoGo task: a NIRS study. Neuroreport.

[21] Ehlis AC, Bahne CG, Jacob CP, Herrmann MJ, Fallgatter AJ (2008). Reduced lateral prefrontal activation in adult patients with attention-deficit/hyperactivity disorder (ADHD) during a working memory task: a functional near-infrared spectroscopy (fNIRS) study. J Psychiatr Res.

[22] Chenxi L, Chen Y, Li Y, Wang J, Liu T (2016). Complexity analysis of brain activity in attention-deficit/hyperactivity disorder: A multiscale entropy analysis. Brain Res Bull.

[23] Takahashi T (2009). Age-related variation in EEG complexity to photic stimulation: a multiscale entropy analysis. Clin Neurophysiol.

[24] Takahashi T (2013). Complexity of spontaneous brain activity in mental disorders. Prog Neuropsychopharmacol Biol Psychiatry.

[25] Stam CJ (2005). Nonlinear dynamical analysis of EEG and MEG: review of an emerging field. Clin Neurophysiol.

[26] Fernandez A, Andreina MM, Hornero R, Ortiz T, Lopez-Ibor JJ (2010). [Analysis of brain complexity and mental disorders]. Actas Esp Psiquiatr.

[27] Yao Y (2013). The increase of the functional entropy of the human brain with age. Sci Rep.

[28] Vaseghi, S. V. *Advanced digital signal processing and noise reduction* (John Wiley & Sons, 2008).

[29] Li X, Cui S, Voss LJ (2008). Using permutation entropy to measure the electroencephalographic effects of sevoflurane. Anesthesiology.

[30] Liang Z (2015). EEG entropy measures in anesthesia. Front Comput Neurosci.

[31] Li X, Ouyang G, Richards DA (2007). Predictability analysis of absence seizures with permutation entropy. Epilepsy Res.

[32] Sokunbi MO (2013). Resting state fMRI entropy probes complexity of brain activity in adults with ADHD. Psychiatry Res.

[33] Lipsitz LA, Goldberger AL (1992). Loss of ‘complexity’ and aging. Potential applications of fractals and chaos theory to senescence. JAMA.

[34] Lipsitz LA (2004). Physiological complexity, aging, and the path to frailty. Sci Aging Knowledge Environ.

[35] Yentes JM (2013). The appropriate use of approximate entropy and sample entropy with short data sets. Ann Biomed Eng.

[36] Chen, X., Solomon, I. & Chon, K. In *Conference proceedings*:… *Annual International Conference of the IEEE Engineering in Medicine and Biology Society. IEEE Engineering in Medicine and Biology Society. Annual Conference*. 4212. 10.1109/IEMBS.2005.1616318 17282095

[37] Bandt C, Pompe B (2002). Permutation entropy: a natural complexity measure for time series. Phys Rev Lett.

[38] Massat I (2012). Working memory-related functional brain patterns in never medicated children with ADHD. PLoS One.

[39] Mattfeld AT (2016). Dissociation of working memory impairments and attention-deficit/hyperactivity disorder in the brain. Neuroimage Clin.

[40] Sohn, H., Lee, W., Kim, I. & Jeong, J. In *World Congress on Medical Physics and Biomedical Engineering 2006*. 1083–1086 (Springer).

[41] Sohn H (2010). Linear and non-linear EEG analysis of adolescents with attention-deficit/hyperactivity disorder during a cognitive task. Clin Neurophysiol.

[42] Li D, Li X, Liang Z, Voss LJ, Sleigh JW (2010). Multiscale permutation entropy analysis of EEG recordings during sevoflurane anesthesia. J Neural Eng.

[43] Baddeley A (2003). Working memory: looking back and looking forward. Nat Rev Neurosci.

[44] Elliott R (2003). Executive functions and their disorders. Brit Med Bull.

[45] Smith EE, Jonides J (1998). Neuroimaging analyses of human working memory. Proc Natl Acad Sci USA.

[46] Cubillo A, Halari R, Smith A, Taylor E, Rubia K (2012). A review of fronto-striatal and fronto-cortical brain abnormalities in children and adults with Attention Deficit Hyperactivity Disorder (ADHD) and new evidence for dysfunction in adults with ADHD during motivation and attention. Cortex.

[47] Shallice, T. *et al*. Executive function profile of children with attention deficit hyperactivity disorder. *Dev Neuropsychol* **21**, 43–71, doi:10.1207/S15326942DN2101_3 (2002). 10.1207/S15326942DN2101_3 12058835

[48] Hu, X. S., Hong, K. S. & Ge, S. S. In *The 2011 International Conference on Advanced Mechatronic Systems*. 435–437.

[49] Monge J (2015). MEG analysis of neural dynamics in attention-deficit/hyperactivity disorder with fuzzy entropy. Med Eng Phys.

[50] McIntosh AR, Kovacevic N, Itier RJ (2008). Increased brain signal variability accompanies lower behavioral variability in development. PLoS Comput Biol.

[51] Schecklmann M (2010). Prefrontal oxygenation during working memory in ADHD. J Psychiatr Res.

[52] Cope M (1988). Methods of quantitating cerebral near infrared spectroscopy data. Adv Exp Med Biol.

[53] Niu HJ (2013). Reduced frontal activation during a working memory task in mild cognitive impairment: a non-invasive near-infrared spectroscopy study. CNS Neurosci Ther.

[54] Gu Y (2016). Empirical mode decomposition-based motion artifact correction method for functional near-infrared spectroscopy. J Biomed Opt.

[55] Ye JC, Tak S, Jang KE, Jung J, Jang J (2009). NIRS-SPM: statistical parametric mapping for near-infrared spectroscopy. Neuroimage.

